# Improving health system access and preparedness in Afghanistan to minimize potential impact of monkeypox: a call for action

**DOI:** 10.1097/JS9.0000000000000137

**Published:** 2023-02-16

**Authors:** Amna Siddiqui, Mohammad Yasir. Essar, Aiman Rija, Michael G. Head, Arash Nemat

**Affiliations:** a Department of Medicine, Karachi Medical and Dental College; b Kabul University of Medical Sciences, Kabul, Afghanistan; c Faculty of Medicine, Dow University of Health Sciences, Karachi, Pakistan; d Clinical Informatics Research Unit, Faculty of Medicine, University of Southampton, Southampton, UK; e Karolinska Institutet, K9 Global Public Health, Stockholm, Sweden

The 2022 monkeypox (MPX) outbreak has seen cases reported in more than 100 countries, with over 81,427 confirmed cases globally as of November 28, 2022^1^. The emergence and sustained transmission across nonendemic settings prompted the WHO to declare it a global health emergency on July 23, 2022.

MPX usually occurs in forested parts of Central and West Africa^2^. There are several potential modes of transmission, typically including via large respiratory droplets, close or direct contact with skin lesions, and possibly through contaminated fomites^2^. Sexual transmission of MPX through seminal or vaginal fluids is not evident yet. Vertical transmission and fetal deaths have been described^3^. Clinical features of the illness include fatigue, fever, chills, and body aches. The incubation period of MPX is 1–2 weeks and the illness can last for around 2–4 weeks^2^. A clinical diagnosis is made on the symptoms and rash, with laboratory tests such as PCR, ELISA, electron microscopy, and immunofluorescent antibody assay can be confirmatory. Once confirmed, smallpox vaccines are ~80% effective^4^. There are experimental antivirals that have been used in some cases for treatment^4^. Hence, to prevent the contraction of the virus strategies like, that is, isolation, hand hygiene, and avoidance of touching contaminated objects and contact with infected animals can be practiced^2^.

Cases have been confirmed across higher and lower-income settings, including in South-East Asia for example in China and India. Afghanistan is a low-income country with a weak healthcare system and significant displacement crisis. This makes communicable disease control difficult, and leaves the country vulnerable to MPX outbreaks^5^,^6^. Thus, the country and international stakeholders must be primed with a preparedness plan around healthcare access that could minimize the impact of any potential MPX outbreak. As of December 2022, there are not yet any confirmed MPX cases within Afghanistan. However, there is also a clear lack of high-quality real-time surveillance data, and thus the true health burdens within Afghanistan are essentially unknown.

Between 2001 and 2021, Afghanistan had worked closely with international stakeholders to invested significantly in reconstructing its health system. This ambition was successful, bringing the coverage of health service from a meagre value of 12% of the population in 2001 to 90% in 2021. Additionally, in early 2021, the government at the time endorsed and integrated two health service packages. These were the Basic Package of Health Services and the Essential Package of Hospital Services. Together they were known as the Integrated Package of Essential Health Services (IPEHS). This IPEHS policy aimed to further expand coverage and scope of health services, moving Afghanistan toward universal health coverage targets^7^.

However, in August 2021, the Taliban invasion and takeover of government has radically changed the political landscape with concerns for the health of the Afghan populations^7^. This meant that the IPEHS was not implemented. Therefore, including the political transition in mid-August, 2021, the temporary halt in funding to the health system from the World Bank will result in the closure of more than 2000 health facilities and unemployment of 25,000 health professionals. This includes 7500 women, and has jeopardized the health and equality gains the country made in past two decades^8^. Subsequently, sustaining the health system becomes a major challenge, exposing one of the poorest nations on the planet to extreme vulnerability.

The orthodox culture of Afghanistan under the Taliban restricts population access, particularly to women and children, to primary healthcare services and impacts the health and well-being of people in Afghanistan. These upstream social determinants of health, such as systemic inequities in the access to basic human rights notably rights and freedoms for women and girls like education and employment, therefore impact upon the opportunity to earn an independent livelihood^9^. . Since mid-2021, more than 1.2 million civilians have been displaced from Afghanistan, including many healthcare providers, further hindering the provision of healthcare services in the country^10^.

Consequently, Afghanistan is poorly placed to address any future MPX outbreaks. Other infections are likely to thrive, including recurrent outbreaks of pandemic coronavirus disease-2019 and vaccine-preventable diseases like poliovirus and measles^11^. This lack of preparedness is enhanced by the presence of many other health challenges, including food insecurity, malnutrition, and child and maternal mortality^12^. Further, stigma and misinformation is a significant MPX issue globally^2^, and there may be real issues with individuals being reluctant to report MPX-like symptoms to a clinic. Health promotion will also be very limited across Afghanistan, and widespread awareness campaigns are virtually nonexistent.

The possibility of MPX spreading to Afghanistan led to concerns arising due to the already prevalent economic crisis in Afghanistan^13^. In June 2022, the Taliban Government announced that Afghanistan would be receiving testing kits for the MPX virus from the WHO^14^. The extent to which these kits have arrived in-country and actually been distributed to health centers is unknown.

The dire needs of this nation include international funds, the supply of vaccines and anti-viral medications. Significant steps are required by the government to establish and implement preparedness policies that minimize the need for response should an outbreak emerge. Policymakers and healthcare providers should raise public awareness of MPX signs and symptoms encourage reporting of suspected cases to a health facility, and provide training to healthcare workers to recognize cases and provide sensitive and appropriate advice. Isolation of suspected cases, contract tracing and surveillance, and ring vaccination protocols can help to curb the virus spread^6^, and thus guidance on disease control should be openly available to healthcare workers and public health teams. As witnessed during the coronavirus disease-2019 pandemic, preparedness around early containment protocols and public awareness, can prove vital in alleviating a health crisis^10^.

Controlling a stigmatized disease like MPX will be difficult in any setting, particular a low-income area where there is displacement and conflict. Given the limited resources available, Afghanistan should therefore prioritize preparedness around preventing any widespread MPX outbreak, with support from international stakeholders.

## Ethical approval

Not applicable. The current article is a correspondence article and no primary data involving human or animal subjects was shared, so an ethical approval was not required.

## Sources of funding

None.

## Author contribution

M.Y.E.: conceptualization. A.S., A.R., M.Y.E., and A.N.: writing original draft. M.G.H.: review and editing.

## Conflict of interest disclosure

The authors declare that they have no financial conflict of interest with regard to the content of this report.

## Research registration unique identifying number (UIN)

None.

## Guarantor

Mohammad Yasir Essar.

## Data statement

None.
